# The Highly Interrelated Morbidity Respiratory Viruses Cause Among Humans and Animals in Mongolia

**DOI:** 10.3390/v17121557

**Published:** 2025-11-28

**Authors:** Maralmaa Enkhbat, Ulziikhutag Batzorig, Nyamaakhuu Dashdondog, Claudia M. Trujillo-Vargas, Davaalkham Dambadarjaa, Gregory C. Gray

**Affiliations:** 1 Division of Infectious Diseases, Department of Medicine, University of Texas Medical Branch, Galveston, TX 77555, USA; eph24e303@gt.mnums.edu.mn (M.E.); ulzi0409@gmail.com (U.B.); nyamaakhuu@mnums.edu.mn (N.D.); cltrujil@utmb.edu (C.M.T.-V.); 2 Mongolian Center for Occupational and Environmental Health, Mongolian National University of Medical Sciences, Ulaanbaatar 14210, Mongolia; davaalkham@mnums.edu.mn; 3 Department of Environmental Health, School of Public Health, Mongolian National University of Medical Sciences, Ulaanbaatar 14210, Mongolia; 4 Department of Microbiology and Immunology, University of Texas Medical Branch, Galveston, TX 77555, USA; 5 Institute for Human Infections and Immunity, University of Texas Medical Branch, Galveston, TX 77555, USA

**Keywords:** Mongolia, respiratory viruses, zoonoses, influenza, epidemiology, review

## Abstract

Mongolia is unique for its cold climate, its large populations of free-roaming livestock, its dense populations of people living in often crowded cities with high air pollution, and its subsequent elevated respiratory virus morbidity among its people and animals. In this narrative review, we examine what is known about this respiratory virus morbidity in Mongolia, organized through the lens of six respiratory virus families: *Orthomyxoviridae*, *Coronaviridae*, *Pneumoviridae*, *Adenoviridae*, *Paramyxoviridae* and *Picornaviridae*. We do so by summarizing published reports regarding respiratory virus morbidity affecting humans or animals in Mongolia. Reports were gathered through a comprehensive review of documents in English language that included peer-reviewed scientific publications, and summary reports from publicly available international health and development organizations. Our review describes the epidemiology and characteristics of specific viruses from these families, describes their transmission and health impacts, and highlights areas where further research and more public health or veterinary health interventions are needed.

## 1. Introduction

Respiratory viruses pose a significant global health threat, particularly in low- and middle-income settings. According to the World Health Organization (WHO) lower respiratory infections accounted for an estimated 344 million new cases and 2.18 million deaths worldwide in 2021, with more than 254,000 of these deaths occurring in low-socio-demographic-index countries [1,2].

Since its emergence in late 2019, the COVID-19 pandemic is estimated to have caused more than 6.9 million deaths globally, placing sustained pressure on health systems across all regions. Prior to COVID-19, seasonal influenza was the predominant viral contributor to global respiratory infection burden, and despite the pandemic, it continues to represent a major source of morbidity and mortality [3,4,5].

**Figure 1:**
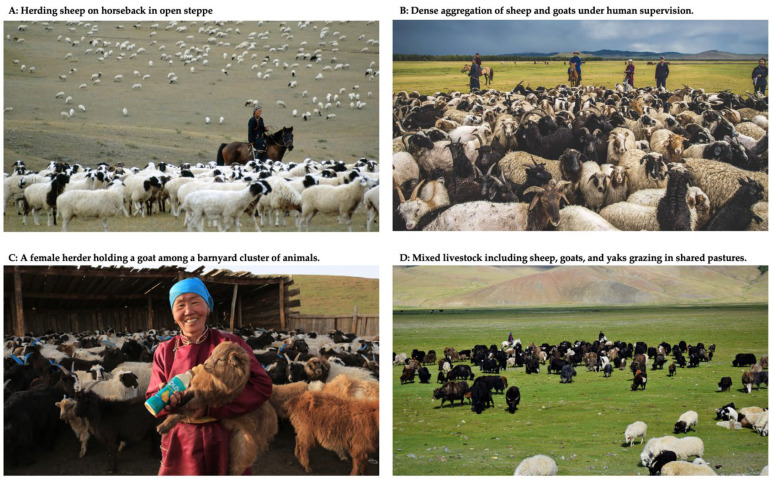
Human–Livestock Interface in Mongolia’s Steppe Ecosystems. As of 2023, Mongolia’s national livestock population exceeded 64 million animals, comprising 29.4 million sheep, 24.6 million goats, 5.47 million cattle, 4.8 million horses, and 473,900 camels [10]. Photographs adapted from Montsame News Agency, Mongolia and the Food and Agriculture Organization of the United Nations (FAO), used under editorial or public domain license.

Within this global context, Mongolia presents a distinct combination of epidemiological, geographical, and environmental risk factors that complicate respiratory virus control. With an estimated human population of 3.546 million in 2024 [6], approximately 50% reside in the capital city, Ulaanbaatar, making it the country’s most densely populated urban center. However, a large portion of the population continues to engage in nomadic herding, living near livestock such as sheep, goats, cattle, camels, and horses (Figure 1). This lifestyle increases the risk of zoonotic spillover [7]. Mongolia also lies along key migratory bird flyways, which makes it susceptible to avian influenza outbreaks. The country’s harsh continental climate, characterized by long, frigid winters and short, dry summers, combined with limited rural health infrastructure, further complicates timely diagnosis, treatment, and prevention efforts [8,9].

Worldwide, approximately two-thirds of emerging infectious diseases originate from animal reservoirs, highlighting the significance of animal-to-human transmission routes in shaping pandemic emergence. The underreporting of animal infections exposes a critical failure to trace the origins of viral transmission, raising concern that despite the availability of treatment and prevention tools, current systems remain inadequate to address the ecological and epidemiological drivers of future pandemics. More than half of documented SARS-CoV-2 animal infections and nearly two-thirds of related deaths were not reported in global surveillance databases [11,12,13,14,15]. Yet, through the integration of multiple independent data sources, researchers identified 35 animal species capable of natural SARS-CoV-2 infection far beyond what was recognized in official records. This expansion of the known host range not only underscores major gaps in early detection but also raises the possibility that other undetected reservoirs may already be enabling future zoonotic spillovers.

Respiratory virus surveillance in animals in Mongolia has revealed several important findings, though data remain limited. Recurrent outbreaks of equine influenza virus (EIV) have been documented among Mongolia’s estimated 2.1 million horses, with a notable 2007 incident involving Bactrian camels, marking the first isolation of EIV in that species and suggesting potential cross-species transmission [16]. In contrast, the 2014 serological study that found no evidence of MERS-CoV in Southern Mongolia Bactrian camels did detect antibodies to bovine coronavirus (BCoV), indicating past exposure to other coronaviruses [17].

Over one million confirmed human cases and 2284 deaths from COVID-19 were reported in Mongolia by early 2025. Respiratory syncytial virus (RSV) also presents a serious pediatric concern, with one study reporting a 37% positivity rate among hospitalized children with lower respiratory infections [18,19]. Influenza is a major seasonal illness, particularly among children and older adults. Environmental stressors such as air pollution from coal burning and winter smog further exacerbate the incidence and severity of pneumonia, which remains the second leading cause of death in children under five [20]. Influenza, caused by influenza viruses, is a major seasonal illness, particularly among children and older adults. Collectively, these intersecting challenges underscore the urgent need for integrated, context specific strategies, especially those informed by One Health principles, to improve surveillance, prevention, and response capacities in Mongolia [21].

In this review we have sought to examine published reports regarding respiratory virus morbidity affecting humans or animals in Mongolia. Our review focuses on six major viral families (*Orthomyxoviridae*, *Coronaviridae*, *Pneumoviridae*, *Adenoviridae*, *Paramyxoviridae* and *Picornaviridae*), that have been found to cause most human respiratory virus epidemics worldwide [22,23]. Through this review we describe the epidemiology and virology characteristics of specific viruses from these families, describe their transmission and health impacts, and highlight areas where further research is needed. In doing so, we also consider Mongolia’s current limitations in viral detection and make recommendations regarding strengthening Mongolia’s laboratory, public health and veterinary health capabilities with a goal of improving national preparedness for future respiratory virus outbreaks.

## 2. Materials and Methods

This narrative review was conducted to examine the burden, transmission, and epidemiological patterns of major respiratory viruses affecting both humans and animals in Mongolia. The review focused on the aforementioned six key viral families. A comprehensive review of documents in English language was undertaken, incorporating national policy frameworks, surveillance records, and peer-reviewed scientific publications. Relevant materials were sourced from publicly accessible databases of international health and development organizations, including the World Health Organization (WHO), the Food and Agriculture Organization of the United Nations (FAO) and the United Nations Development Programme (UNDP), the United Nations Human Settlements Programme (UN-Habitat).

In parallel, a structured literature search was carried out using electronic databases including PubMed, Scopus, Web of Science, and Google Scholar. The search strategy applied Boolean operators to combine keywords relating to respiratory viruses and Mongolia. Search terms included combinations such as (“respiratory viruses” OR “respiratory infections”) AND (“Mongolia”) AND (“humans” OR “animals” OR “livestock” OR “zoonotic”) AND (names of specific viruses or families such as “influenza”, “coronavirus”, “RSV”, “adenovirus”, “paramyxovirus”, or “rhinovirus”). Articles were included if they presented primary or secondary data on virus detection, surveillance, outbreaks, prevalence, or transmission in Mongolia, regardless of publication year. Only studies published in English and directly related to the Mongolian context were included.

All records retrieved through the search strategy were screened in three stages: title review, abstract review, and full-text assessment. Duplicates were removed prior to screening. At each stage, eligibility was determined based on the predefined inclusion and exclusion criteria. The overall selection approach followed PRISMA-style principles to ensure transparency and reproducibility.

The number of human and animal virus types/subtypes shown in Table 1. For humans, 20 unique virus types/subtypes were identified based on surveillance and epidemiological reports. For animals, 278 unique virus types/subtypes were identified, largely due to extensive subtype diversity in avian influenza viruses and livestock viral pathogens. Each subtype was counted once per viral family based on official classifications reported in the source documents.

The following sections summarize the impact of specific viruses within each family, including their effects on human and animal populations, epidemiological patterns, virus types and subtypes, surveillance data, outbreak records, and public health interventions.

### 2.1. Study Team and Screening Process

This literature review was first undertaken by a three-member research team (M.E., U.B., and U.B.). Two reviewers independently screened each record at the title, abstract and full text stages. Any disagreements during the selection or extraction phases were resolved through group discussion with senior authors (G.C.G. and D.D.). All authors contributed to analysis and synthesis of the final findings.

### 2.2. Inclusion Criteria

1. Publications reporting data on respiratory viruses circulating in Mongolia.
2. Records involving humans, animals, or both.
3. Publications written in English and accessible in full text.

### 2.3. Exclusion Criteria

1. Studies unrelated to Mongolia or that did not include Mongolian data.
2. Documents that did not involve any of the six viral families.
3. Conference abstracts without full text, commentaries, news articles, and editorials.
4. Non-English sources or reports lacking accessible full text.
5. Studies focusing solely on non-respiratory pathogens.

## 3. Results

Below we preface our review findings for each viral family with a summary of that viral family’s characteristics.

### 3.1. Orthomyxiviridae

The *Orthomyxoviridae* family includes viruses with enveloped, spherical or pleomorphic virions that contain a segmented, negative-sense single-stranded RNA genome. The genome typically comprises six to eight segments, depending on the viral genus. These viruses infect a broad range of vertebrate hosts, including humans, birds, swine, horses, and marine mammals. The family is classified into several genera, with the most clinically relevant being Alphainfluenzavirus (influenza A virus), Betainfluenzavirus (influenza B virus), Gammainfluenzavirus (influenza C virus), and Deltainfluenzavirus (influenza D virus) [24].

Among them, influenza A viruses are particularly notable for their zoonotic potential and ability to undergo genetic reassortment, leading to the emergence of novel subtypes capable of causing global pandemics. Transmission primarily occurs through respiratory droplets, aerosols, and contact with contaminated surfaces, and these viruses are responsible for seasonal epidemics and occasional outbreaks in both human and animal populations.

#### 3.1.1. Impact of Influenza on Mongolia’s Human Population 

Influenza has imposed a significant disease burden on the Mongolian population, disproportionately affecting vulnerable groups such as children under five years of age, pregnant women, and individuals with chronic health conditions. During the 2009 H1N1 influenza A virus pandemic, Mongolia reported 29 confirmed human deaths, corresponding to a mortality rate of 1.0 per 100,000 population between 12 October 2009 and 31 January 2010 [25]. The crude case fatality rate among hospitalized patients was 2.2%. The highest mortality was observed among young children and middle-aged adults. Furthermore, more than 60% of fatal cases involved individuals with underlying medical conditions, particularly pregnancy, cardiovascular disease, and chronic liver disease, which were found to be five to fifty times more common among fatalities compared to national averages [25].

In several surveillance and cohort studies, children under two years of age accounted for most influenza-like illness (ILI) and severe acute respiratory infection (SARI) cases, as well as the highest rates of hospitalization. One nationwide study found that children in this age group experienced the highest SARI-related mortality, with rates ranging from 15.8 to 54.0 deaths per 100,000 population annually [19,26,27]. Additionally, pregnant women were identified as a high-risk group, with comorbidities increasing their likelihood of developing ILI. These findings confirm that the impact of influenza in Mongolia is concentrated among specific high-risk populations, reinforcing the importance of targeted public health interventions.

#### 3.1.2. Epidemiology

Influenza transmission in Mongolia follows a clearly defined seasonal pattern, with peak activity typically occurring during the winter months from December to February. Studies have reported consistent seasonal waves of influenza-like illness (ILI) and severe acute respiratory infection (SARI), with increased incidence during colder periods likely influenced by environmental conditions, indoor crowding, and air quality.

Transmission within households and schools plays a central role in the spread of influenza in Mongolian communities. A large prospective cohort study identified children aged 1–4 years as key drivers of household and community transmission. During an influenza A (H3N2) outbreak in 2010–2011, this age group exhibited the highest attack rate, reaching 20.4%. The household secondary attack rate was 5.7% overall, and substantially higher among younger household members. Preschool and school settings were identified as key nodes of repeated transmission, suggesting that children not only acquire but also amplify the spread of infection within households and broader community settings [28].

One study conducted in Ulaanbaatar demonstrated a synergistic relationship between air pollution and viral exposure in driving influenza-related hospital visits. Fine particulate matter (PM1, PM2.5), ozone, and nitrogen dioxide levels were shown to amplify the effects of viral activity, particularly during cold months. These effects were strongest among children under five, who experienced significantly higher rates of hospitalization in response to concurrent air pollution and viral exposure. Together, these findings illustrate that influenza transmission in Mongolia is shaped not only by traditional epidemiological dynamics but also by complex environmental and social determinants [29].

#### 3.1.3. Virus Types and Subtypes

Mongolia has experienced the circulation of a diverse range of influenza virus types and subtypes over the past several decades, reflecting both regional and global trends. The most commonly reported influenza A subtypes include seasonal A (H1N1), A (H3N2), and pandemic A (H1N1)pdm09, with outbreaks of each subtype documented through national surveillance data and independent studies. Seasonal influenza B viruses have also played a significant role in disease burden, with both the B/Victoria and B/Yamagata lineages detected [25,30,31].

During the 2009 influenza A (H1N1)pdm09 pandemic, the virus rapidly spread across the country. Seroprevalence studies conducted in Selenge aimag (province) found that approximately one-fourth of the population had been infected during the first wave of the pandemic. Laboratory-confirmed cases were highest among young children, reflecting their susceptibility and central role in transmission [31].

Molecular studies have provided further insights into the genetic characteristics of influenza viruses circulating in Mongolia. Sequencing of historical isolates collected between 1985 and 1991 revealed that several influenza A (H1N1) strains were closely related to reassortant vaccine strains (PR8 × USSR/77) that had been used in earlier national immunization campaigns. These findings suggest that vaccine-derived lineages may have persisted in the population, particularly in areas with limited population density and reduced viral evolution pressure. Some Mongolian strains showed slower mutation and evolutionary rates compared to those isolated from more densely populated regions globally, possibly due to the country’s ecological and demographic characteristics [32].

More recent molecular surveillance has confirmed the presence of influenza B lineages consistent with global patterns. Between 2013 and 2017, B/Victoria clade-1A and B/Yamagata clade-3 viruses were isolated from clinical cases, with confirmed genetic compatibility to the vaccine strains in use during that period. However, some isolates demonstrated reduced susceptibility to neuraminidase inhibitors, and a novel mutation (G104R) was detected, suggesting the potential for emerging antiviral resistance in local strains. This highlights the need for continued genomic surveillance and drug susceptibility testing to monitor viral evolution in the Mongolian context [30].

#### 3.1.4. Surveillance Data and Outbreaks

Influenza surveillance in Mongolia has first established in the 1970s. Influenza B virus was predominant during the 2007–2008 and 2011–2012 seasons, while seasonal A (H1N1) circulated in 2008–2009. A (H3N2) dominated during 2010–2011, followed by the emergence of pandemic A (H1N1) pdm09 in 2009–2010, which caused widespread community transmission and increased disease severity [25,30,33].

Serological studies conducted in provinces such as Selenge confirmed that the 2009 pandemic affected approximately one-fourth of the population, as determined by hemagglutinin inhibition and microneutralization assays. The age group with the highest seroconversion rates was children aged 2–9 years [31].

Across five influenza seasons between 2013 and 2018, the estimated annual ILI incidence ranged from 1279 to 2798 cases per 100,000 population, while SARI incidence varied from 81 to 666 per 100,000. The burden was disproportionately high in children under five, who accounted for 67% of all ILI cases and 79% of all SARI cases reported. Among laboratory-tested specimens, annual influenza positivity rates reached up to 30% for ILI and 31% for SARI, particularly in children under 15 years of age [18].

In addition to routine surveillance, targeted outbreak investigations have provided valuable insights into transmission dynamics. The surveillance and outbreak data collectively demonstrating resource-constrained capacity of Mongolia’s health system to monitor influenza activity and support response planning [28].

#### 3.1.5. Public Health Interventions

Public health responses to influenza in Mongolia have evolved alongside expanding surveillance networks, targeted research, and persistent healthcare challenges. Multiple studies emphasize the critical need for timely antiviral treatment, robust surveillance systems, and context-specific vaccination strategies to manage influenza and related viral infections effectively [19,25,26,27,31,34].

During the 2009 A (H1N1) pdm09 pandemic, hospital-based surveillance highlighted that most fatal cases did not receive antivirals within the first 48 h of symptom onset. This delay reduced treatment effectiveness and revealed key limitations in emergency response capacity. A community-based research among pregnant women and infants in Baganuur (one of nine duureg (districts) of the Mongolian capital of Ulaanbaatar) revealed substantial disease burdens from both influenza and RSV, reinforcing the urgency of early detection and targeted protection for vulnerable groups [34].

Nationally, Mongolia’s influenza surveillance infrastructure has grown substantially, now encompassing over 150 sentinel sites. This network has improved the detection of ILI and SARI trends, including seasonality and age-group distribution, with young children bearing the highest burden of illness [19,27]. Data from five influenza seasons indicate that routine vaccination, particularly among children under five, could meaningfully reduce disease severity and hospitalizations. However, implementation of a national vaccination strategy remains limited.

The country currently lacks sufficient virological infrastructure to detect novel or emerging respiratory viruses, especially those that may elude commercially available diagnostics developed for other populations.

#### 3.1.6. Influenza’s Impact upon Mongolia’s Animal Population

1. Domestic animals

Mongolia’s livestock-based economy [35] relies on domestic animals, including horses, camels, and poultry. Recurrent influenza outbreaks have caused substantial morbidity among these species, particularly equines. Equine influenza A (H3N8) virus has shown sustained circulation in Mongolian horses, contributing to repeated epizootics over the past decades. For example, during the 2011 outbreak, widespread infection among free-ranging horse herds in multiple provinces was confirmed. Viral isolates obtained from symptomatic horses were closely related to H3N8 strains circulating in Central Asia, particularly those from earlier outbreaks in 2007 and 2008, pointing to persistence of a specific viral lineage in the region [35].

Phylogenetic analysis of H3N8 viruses isolated from horses in Mongolia and those imported into Japan further affirmed the presence of two distinct clades of the Florida sublineage, with Mongolian isolates belonging to clade 2. Interestingly, antigenic analysis revealed minimal variation across isolates, indicating evolutionary stability in hemagglutinin despite widespread geographical distribution [33,36].

2. Wild birds and wildlife

Mongolia is a critical region for migratory bird populations, intersecting multiple flyways for species traveling between Asia, Europe, and Africa. This ecological interface has made it a hotspot for avian influenza viruses (AIVs), including both low-pathogenicity avian influenza viruses (LPAIVs) and highly pathogenic avian influenza viruses (HPAIVs). Repeated isolations of HPAI H5N1, H5N6, and H5N8 subtypes from wild birds have been documented [37,38,39,40,41,42].

Mongolian wildlife beyond birds also appears to be involved in influenza ecology. Notably, serological studies in Mongolian wild ass or Gobi khulan (*Equus hemionus*) detected antibodies to multiple hemagglutinin subtypes, including H1, H3, H5, H7, H8, and H10 [43]. Given the sympatry between domestic and wild equids, these findings raise the possibility that khulan may serve as underrecognized reservoirs for influenza viruses in shared landscapes.

3. Zoonotic interfaces and spillover risks.

The ecological convergence of domestic animals, migratory birds, and humans in Mongolia presents significant risks for interspecies transmission of influenza viruses. Although direct zoonotic transmission to humans has not been widely reported, serological evidence indicates potential spillover events. A study conducted among Mongolian adults with occupational animal exposure found low-level seroreactivity to avian and equine influenza viruses, although none of the titers reached diagnostic thresholds [44,45].

The discovery of H3N8 virus in camels [16] and H5N1 antibodies in horses [46] raises concerns about novel reassortment events. The presence of both avian and equine viruses in a single host species creates a permissive environment for genetic mixing, especially given the lack of systematic animal vaccination and biosecurity protocols. Mongolia’s unique animal husbandry practices, which favor close contact between species, amplifying the risk of novel virus emergence with potential for zoonotic spillover.

#### 3.1.7. Epidemiology

1. Transmission pathways

The transmission of animal influenza viruses in Mongolia occurs through a complex web of interspecies interactions, environmental overlap, and ecological migration patterns. Wild birds play a central role in spreading avian influenza viruses (AIVs), particularly via fecal–oral transmission in water bodies shared by domestic and migratory species. Environmental fecal sampling revealed the presence of AIVs across multiple seasons, particularly during autumn migrations, highlighting water as a significant medium for viral transmission in wild bird populations [47,48].

**Figure 2:**
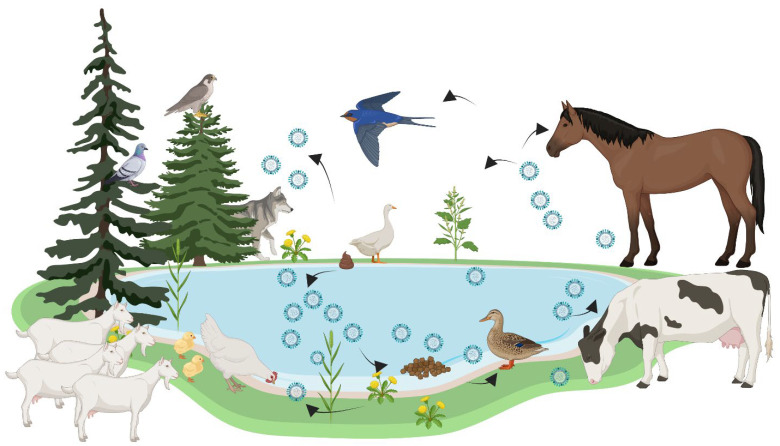
Influenza A transmission pathways among wildlife in Mongolia Watersheds are an important route of viral transmission in Mongolia. They serve as common gathering points for migratory and domestic birds, livestock raised for human consumption, and carnivores, which increases the likelihood of oral–fecal exposure to the virus. Created in BioRender. Trujillo Vargas, C. (2025) https://BioRender.com/83bd1jn.

Migration contributes significantly to the introduction and dispersal of highly pathogenic avian influenza (HPAI) strains (Figure 2). For instance, satellite tracking of whooper swans confirmed long-distance transmission of H5N1 from China to Mongolia during seasonal migration, with genetic sequencing showing identical strains at both endpoints [49]. Such patterns indicate that wild birds do not just maintain viral circulation locally, but also act as international vectors introducing novel strains.

2. Geographical and seasonal patterns

The distribution of influenza outbreaks and viral prevalence in Mongolia exhibits clear geographic and seasonal trends. Outbreaks of HPAI and EIV are more commonly detected in central and western Mongolia, such as Töv, Khentii, and Govi provinces, which serve as migratory stopovers and breeding grounds for numerous avian species [37,39,41]. Lakes and wetlands in these regions act as convergence zones where waterfowl from different flyways congregate and facilitate cross-transmission.

In fall, virus prevalence in wild bird fecal samples rose as high as 2.5% compared to below 1% during early summer. These seasonal patterns have direct implications for timing surveillance and targeted interventions.

3. Environmental and ecological factors

The nomadic pastoralism that dominates rural Mongolia contributes to influenza transmission by increasing interactions between livestock, wildlife, and the natural environment. Horses and camels are often free-ranging, moving across vast open pastures, often overlapping with habitats of migratory birds or wild equids. This interface increases opportunities for interspecies transmission [7,16,43].

Wetland ecosystems serve as critical reservoirs for influenza viruses. These areas, such as Khunt (Zavkhan province, Mongolia), Ganga (Sukhbaatar province, Mongolia), and Doroo (Dornod province, Mongolia) Lakes, support seasonal congregation of migratory waterfowl and have been repeatedly associated with the detection of HPAI viruses [37,38,41]. Environmental sampling in these habitats, including fecal collection and carcass testing, has provided essential data for tracking influenza spread and diversity [47,48].

The environmental persistence of viruses in cold climates, combined with limited infrastructure for veterinary disease monitoring, complicates early detection and rapid response. In several cases, highly pathogenic strains such as H5N1 and H5N6 were identified only after mass mortality events had already occurred among birds, indicating that silent transmission had been ongoing [38,41,42].

Moreover, ecological factors like population density of birds, overlapping flyways, and breeding cycles play a key role in the annual influx of novel strains into Mongolian ecosystems. Mongolia’s strategic position at the junction of East Asian-Australasian, Central Asian, and West Pacific migratory flyways renders it particularly vulnerable to introductions of genetically diverse viruses [38,47,48].

4. Virus types and subtypes relevant to Mongolia

Molecular epidemiology has revealed complex patterns of viral evolution and reassortment in Mongolia. Viruses isolated from birds and mammals often show genetic similarity to strains circulating in neighboring countries, emphasizing the role of transboundary transmission. Genetic analysis of H5N1 strains isolated in 2009, for instance, demonstrated clustering with viruses from South Korea and China, indicating regional spread via migratory birds [38].

Reassortment events were observed among viruses isolated from gulls, ducks, and camels, with some strains combining gene segments from Eurasian and North American lineages, illustrating the mosaic nature of viral genomes in regions of flyway convergence [7,38]. A camel isolate revealed genetic similarity to human-origin viruses, supporting the hypothesis of human-to-camel or environment-to-camel transmission [16,50].

Ducks infected with the MON3 H5N1 strain demonstrated severe neurovirulence, with mortality linked to replication in brain tissue. This suggests specific gene combinations, such as PB2 and NP, are key drivers of pathogenicity in certain hosts [42].

Collectively, these molecular findings underscore the dynamic and evolving nature of influenza viruses in Mongolia. Reassortment, host adaptation, and cross-species infections continue to challenge the understanding and control of influenza epidemiology in animal populations.

#### 3.1.8. Surveillance Data and Outbreaks

Influenza virus surveillance in Mongolia has undergone significant development over the past two decades. Given its geographical position at the crossroads of multiple migratory bird flyways, and the nomadic animal husbandry system that promotes high contact rates between domestic animals and wildlife, Mongolia represents a unique ecological niche for the emergence and spread of diverse influenza viruses. Surveillance activities have focused on domestic animals, wild birds, and, in select cases, camels and equids. The majority of these efforts have been conducted through fecal sampling, nasal swabs, and environmental surveillance, alongside molecular diagnostics and phylogenetic analyses to characterize viral subtypes and trace origins of outbreaks [37,39,47,48,51].

Sentinel surveillance strategies have been employed to detect both low-pathogenic and highly pathogenic avian influenza viruses in wild birds. In parallel, active surveillance has also been conducted for equine influenza virus among domestic and wild equids [7,35,36,43,52]. These surveillance systems have not only documented the prevalence of known subtypes but have also led to the identification of novel reassortant viruses and uncharacteristic host–virus dynamics, including spillover events into camels and wild ungulates [16,43,46,50].

#### 3.1.9. Animal Health Interventions

Mongolia’s capacity to respond effectively to animal influenza virus outbreaks remains limited despite being positioned at a critical intersection of migratory bird flyways and a country with a large, highly mobile domestic animal population. While evidence of infection, transmission, and reassortment of various influenza A virus subtypes in domestic and wild animals has been well documented, interventions to mitigate their impact appear fragmented, insufficiently resourced, and reactive rather than preventive.

Influenza outbreaks among Mongolian horses, especially those caused by equine influenza A H3N8, are reported approximately every decade and critically disrupt nomadic livelihoods and national economy. Surveillance during the 2011 epizootic highlighted that while an active monitoring system was in place, only three influenza-positive specimens were successfully isolated among 745 horses sampled, and most of the horses swabbed exhibited no clinical symptoms. This raises questions about both the sensitivity of the diagnostic tools and the broader effectiveness of the animal surveillance infrastructure in detecting subclinical or emerging strains [35].

Moreover, repeated serological and virological investigations have shown the continuous circulation of influenza viruses among horses, Bactrian camels, and even wild equids such as Mongolian wild horses. Despite this, none of the herder households in a 2016 to 2017 surveillance study had vaccinated their horses against equine influenza, even after five documented epizootics since 1970 [7]. The lack of vaccination campaigns represents a critical intervention gap in national veterinary strategy. Furthermore, a detected horse-to-camel transmission event of equine influenza A virus illustrates the zoonotic potential and fluidity of viral host ranges, which makes the case for expanding vaccination not just among horses but also among other susceptible domestic animals [16].

Avian influenza virus monitoring in Mongolia has been more structured in comparison. Environmental fecal sampling conducted from 2009 to 2023 has been a cost-effective and scalable approach to detect low-pathogenicity avian influenza viruses. Still, high pathogenicity subtypes, including H5N6 and H5N1, have only been discovered following wild bird mortality events or through isolated surveillance episodes [37,40,41,53]. This reactive mode of detection has major consequences. By the time mortality is observed, viruses have likely already disseminated across domestic and wild animal populations, increasing the likelihood of further reassortment and cross-species transmission.

Efforts to characterize viral subtypes and clades have been commendable, particularly with advanced gene sequencing and phylogenetic analyses that allowed identification of clade 2.3.4.4h H5N6 viruses [41,53]. However, while diagnostic precision has improved in research contexts, the translation of this information into real-time, field-level interventions remains limited. There is no indication from the literature that Mongolia maintains any standardized field-deployable molecular surveillance protocols outside of research-led efforts. Similarly, detection of antibody titers in wild birds is an important signal of past exposure, but without a parallel in systematic vaccination or biosecurity policy, such information remains underutilized [54].

Furthermore, there is a noticeable absence of targeted control measures in regions identified as high-risk. For instance, studies have revealed repeated H5N1 outbreaks in migratory waterfowl at Khunt, Erkhel, and Ganga Lakes, yet there is no mention of localized restrictions, buffer zones, or even public advisories in these ecologically sensitive areas [37,49]. Such absence of zonal intervention strategies suggests a disconnect between surveillance outputs and operational policy.

For example, documented spillover of H5N1 into horses [46], identification of novel mutations in circulating avian strains [43,53], and the detection of reassortant viruses with pandemic potential [55] all demand a coordinated intersectoral response. Yet, the findings are discussed in siloed studies rather than being integrated into a comprehensive response mechanism.

Crucially, the absence of sustained vaccination campaigns across high-risk animal populations is perhaps the most glaring intervention gap. Equine influenza vaccines based on the Florida clade 2 strain are available and have been recommended in multiple reports, but implementation has been virtually nonexistent in Mongolia [35,52]. Similarly, although antigenic matching of influenza B virus lineages to vaccine strains has been confirmed in the past [37], no systematic poultry or avian vaccination program has been documented.

Moreover, Mongolia has not established emergency response protocols for animal influenza outbreaks, including protocols for animal movement restrictions, quarantine, or mass culling in the event of a high-mortality outbreak. This lack of preparedness was evident in the handling of H5N6 and H5N1 cases identified in wild birds and horses. While the detection of zoonotic subtypes should have triggered urgent interventions, there is no indication that preventive actions were taken to reduce cross-species spillover [46,53].

The final layer of vulnerability is tied to knowledge translation and communication. Surveillance data, when available, are rarely disseminated in a timely manner to herders, veterinary officers, or border officials. The lack of formal training programs in emerging zoonoses and viral diagnostics for local veterinarians has likely further limited the effectiveness of field response. Surveillance in wild equids, for instance, was conducted with sophisticated techniques, yet it is unclear if these results have influenced any changes in surveillance practice or public awareness campaigns [43].

### 3.2. Coronaviridae

*Coronaviridae* is a family of enveloped RNA viruses that infect both humans and animals. The family includes viruses responsible for respiratory, gastrointestinal, hepatic, and neurological diseases. It is divided into two subfamilies: *Orthocoronavirinae*, which infects mammals and birds, and *Letovirinae*, which is less well understood. Human-infecting members include SARS-CoV, MERS-CoV, and SARS-CoV-2, all belonging to the *Betacoronavirus* genus. These viruses can cause diseases ranging from mild colds to severe pneumonia. Animal coronaviruses are widespread and infect species such as bats, camels, horses, swine, cattle, and birds. Cross-species transmission is a defining feature of this virus family, often driven by mutation and recombination. The large genome and the ability to recombine contribute to their emergence as zoonotic threats. The global impact of SARS-CoV-2 has highlighted the significance of *Coronaviridae* in both public and veterinary health, reinforcing the need for integrated One Health approaches to monitor and control outbreaks [56].

#### 3.2.1. Epidemiology

1. Transmission routes

The emergence and global spread of SARS-CoV-2 posed a significant challenge for countries worldwide. Mongolia, a lower-middle-income country with a sparse population and extensive borders, exhibited a unique epidemiological trajectory shaped by early and rigorous public health interventions. These strategies initially helped delay widespread transmission, allowing Mongolia to report no local cases until November 2020 despite the virus’s global circulation beginning in early 2020 [57].

By the end of 2020, Mongolia had one of the lowest SARS-CoV-2 seroprevalence rates globally. A national survey conducted between October and December 2020 found that just 1.5 percent of the population had detectable SARS-CoV-2 antibodies, adjusted for population weight and test performance [57]. This exceptionally low figure underscored the success of early governmental containment efforts, including border closures, school shutdowns, and mask mandates, all implemented before any confirmed local transmission [57,58].

**Figure 3:**
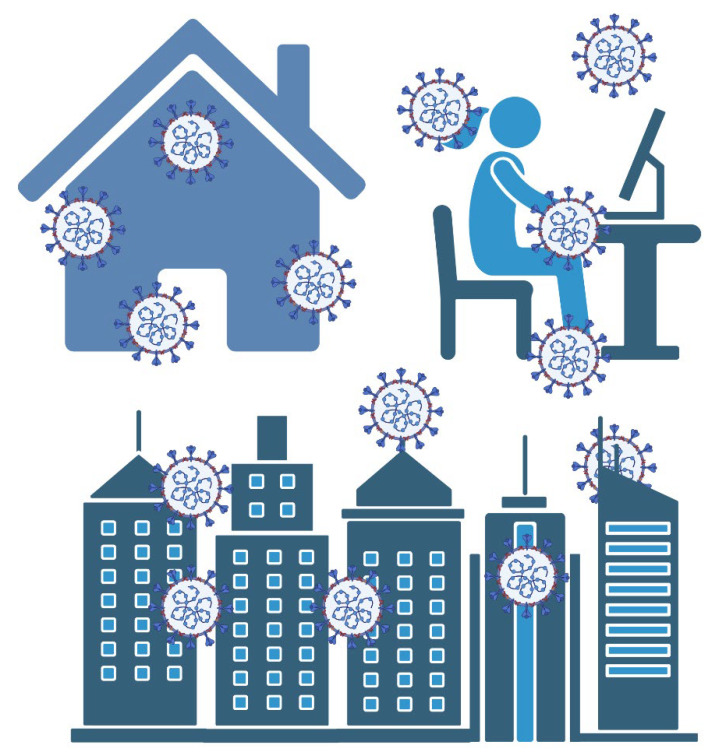
SARS-CoV-2 disparity in transmission. Household and workplaces in Ulaanbaatar were the epicenter of viral spreading during the COVID-19 pandemic. Created in BioRender. Trujillo Vargas, C. (2025) https://BioRender.com/oivu8ct.

However, the landscape shifted dramatically in 2021. Transmission increased in large cities in Mongolia (Figure 3) with evidence that transmission was associated with exposures where people mixed such as workplaces and households. With the introduction of mass vaccination campaigns starting in February 2021, Mongolia’s public health strategy evolved from containment to mitigation. By December 2021, national seroprevalence had soared to 82.3%, reflecting both widespread vaccination and extensive community-level transmission among the unvaccinated population. Among the unvaccinated, approximately 64.5% had been infected, indicating a substantial rise in natural infections as protective measures were eased [59].

Despite the significant rise in infections in 2021, Mongolia maintained a relatively low infection fatality rate of 0.1% [59]. The low mortality figures may be attributed to a younger population structure, early case identification, and strong health system mobilization during initial outbreaks. Nevertheless, hospital capacity was periodically strained during surges, and testing backlogs hindered real-time response [60].

The rapid vaccine rollout in 2021 further shaped the impact on Mongolia’s population. The national program utilized four vaccine platforms: Pfizer-BioNTech (BNT162b2), AstraZeneca (ChAdOx1-S), Sinopharm (BBIBP-CorV), and Sputnik V (Gam-COVID-Vac). A study comparing post-vaccination antibody responses among healthy individuals found significant differences in immunogenicity across vaccine types. The mRNA vaccine (Pfizer-BioNTech) elicited the highest levels of neutralizing antibodies, whereas inactivated vaccines such as Sinopharm induced the lowest [60,61,62]. This variability in antibody response likely contributed to differences in breakthrough infection risk and may have impacted the scale of reinfection and disease severity at the population level [60].

Breakthrough infections became a major concern as cases surged in mid-2021. Among hospitalized patients with symptomatic breakthrough infections, 94.6% were found to be infected with the Alpha variant and 5.4% with the Delta variant. A majority of these cases had received the BBIBP-CorV vaccine, raising concerns regarding the vaccine’s protective capacity against emerging variants. The antibody neutralization assays corroborated these concerns, showing that vaccines based on the Washington strain conferred limited neutralizing activity against newer variants, including Delta [60].

Mongolia’s general population experienced psychological and economic effects from the prolonged pandemic. A cross-sectional study documented elevated levels of depression, anxiety, and stress among Mongolian citizens during strict lockdowns, particularly after the first wave of community transmission. Factors contributing to mental health deterioration included uncertainty, financial strain, limited mobility, and lack of social interaction. Individuals with lower perceived social support were more likely to report severe mental health symptoms [63].

Healthcare workers, in particular, bore a disproportionate psychological burden. A study among frontline Mongolian healthcare professionals revealed a high prevalence of anxiety (70.2%), depression (52.3%), and stress (35.8%) during COVID-19 peaks. Long working hours, insufficient rest, and fear of infection were key contributors. Moreover, inadequate protective equipment and unclear policies on risk communication exacerbated occupational stress [64].

In terms of occupational protection, the majority of Mongolian healthcare workers supported mandatory COVID-19 vaccination for healthcare personnel. A survey found that 93.7% agreed with this policy, though actual vaccine willingness was slightly lower at 67.2%. Hesitancy was associated with lack of confidence in vaccine efficacy and concerns about side effects. Among younger workers and those in tertiary hospitals, willingness to vaccinate was notably lower [65].

The government undertook extensive testing and surveillance programs, including a mass testing strategy in Ulaanbaatar termed the “One door-one test” (ODOT) approach. This campaign aimed to identify asymptomatic carriers during a February 2021 lockdown but was criticized for its cost inefficiency and poor design. The program tested one adult per household across 420,000 homes but detected only 131 cases, many of which were close contacts. Critics argued that the approach created a false sense of security, diverted resources from essential services, and failed to consider within-household transmission dynamics [66].

Air quality in Ulaanbaatar showed notable improvement during COVID-19 lockdowns due to reduced human activity and traffic. Levels of nitrogen dioxide, PM10, and PM2.5 decreased substantially during lockdown months compared to historical baselines. Conversely, sulfur dioxide levels increased, likely due to higher indoor coal burning in the absence of outdoor movement [67]. These environmental shifts reveal both unintended positive and negative impacts of pandemic restrictions on urban health.

Overall, the population-wide effects of SARS-CoV-2 in Mongolia were shaped by a combination of early preparedness, variable vaccine performance, emergence of new viral variants, and socio-economic vulnerabilities. While mortality was relatively contained, the psychological, occupational, and economic burdens were significant. The variation in vaccine-induced immunity, particularly the lower neutralization efficacy of certain vaccine types, presents ongoing public health challenges. Likewise, the temporary gains in air quality highlight the need to integrate environmental sustainability with pandemic recovery.

2. Vulnerable groups and risk

A longitudinal seroepidemiological study conducted through 2021 identified occupational and demographic disparities in exposure and seroconversion. Health workers were consistently at higher risk for COVID-19 diagnosis across all survey rounds. Adults over 20 years of age and males had significantly higher odds of seroconversion during the vaccine rollout phase, with odds ratios of 12.7 and 1.7, respectively [59]. These findings indicate a need for targeted occupational protections and risk communication strategies, particularly for frontline healthcare professionals.

Indeed, the pandemic imposed a severe mental health burden on healthcare workers. In a cross-sectional study of 965 healthcare professionals, depression (52.3%), anxiety (70.2%), and stress (35.8%) were highly prevalent [64]. Long working hours and the rapid expansion of healthcare service demands under pandemic conditions contributed to psychological distress. These stressors likely affected infection control practices and the ability of the healthcare system to manage patient loads effectively.

3. Vaccination and immunity

Following the initiation of a national vaccination campaign in February 2021, Mongolia quickly reached high levels of vaccine coverage. By July 2021, 60% of the population was fully vaccinate [59], a milestone achieved through rapid deployment of four vaccine types: Pfizer/BioNTech, AstraZeneca, Sputnik V, and Sinopharm [60,61,62]. However, despite high coverage, outbreaks continued, particularly in mid-2021. In one study, breakthrough infections among vaccinated individuals were primarily caused by the Alpha variant (94.6%), with the Delta variant accounting for a small minority (5.4%) [60].

Antibody response analysis showed marked differences among the vaccine types. Pfizer/BioNTech generated the highest neutralizing antibody levels, followed by AstraZeneca, while Sinopharm and Sputnik V resulted in significantly lower titers [61,62,68]. This variability had direct epidemiological consequences, as weaker immune responses correlated with higher rates of breakthrough infections during later waves.

Moreover, the antibody persistence varied significantly. Six months after vaccination or natural infection, BNT162b2 (Pfizer) retained robust antibody levels, whereas antibodies from other vaccines declined more sharply [68]. The disparity in immune protection contributed to reinfection risks and required consideration of booster strategies, especially for those who received inactivated or adenoviral vaccines.

4. Social and behavioral influences

Epidemiological dynamics were also influenced by public attitudes and behavioral responses. A nationwide survey revealed that vaccine hesitancy was significantly associated with reliance on social media as a primary information source, while individuals accessing government websites had higher vaccine acceptance [69]. These findings illustrate the impact of communication strategies on vaccination uptake and highlight the need for centralized, evidence-based messaging.

The mental health burden extended beyond healthcare workers to the general population. In a sample of 344 participants, high rates of depression, anxiety, and stress were observed during lockdowns [63]. Those with limited social support networks were particularly vulnerable, illustrating how psychosocial factors intersect with epidemiological outcomes in times of public health crises.

5. Diagnostic capabilities and laboratory gaps

While Mongolia responded quickly with mass testing strategies such as the One-Door-One-Test campaign, critical evaluations have questioned their efficiency and evidence base. The campaign, intended to test one adult from each of the 420,000 households in Ulaanbaatar, detected only 131 cases at a cost of over 6 million USD [66]. The campaign may have yielded a false sense of security and did not accurately capture prevalence due to the use of non-representative samples within households.

On a broader scale, Mongolia’s national capacity for molecular diagnostics and genomic surveillance remains limited. A molecular epidemiology study conducted using nanopore sequencing found delayed introduction of the Alpha and Delta variants compared to global trends [70]. While the delay could be attributed to effective public health measures, the study also highlighted the absence of a systematic sequencing infrastructure prior to 2020. This lack of capacity to detect and monitor variant circulation in real time puts Mongolia at a disadvantage in identifying emerging viral threats.

Mongolia is currently ill-prepared to respond to novel viral agents that may arise within or cross its borders. This vulnerability poses a national security concern in the context of biodefense and highlights an urgent need for investment in genomic surveillance infrastructure and training. Although some initial steps have been taken, such as the implementation of nanopore sequencing in research contexts [70], the scale, integration, and sustainability of such programs remain unclear. There is no consistent national system to link sequencing, clinical data, and epidemiological outcomes.

Environmental and policy contexts have also shaped transmission dynamics. Lockdown policies implemented during the pandemic significantly altered human behavior and even environmental metrics. For example, three sequential lockdowns in Ulaanbaatar led to substantial declines in air pollutants such as PM2.5 and NO2, but sulfur dioxide levels increased [67]. These fluctuations have indirect epidemiological implications, as poor air quality has been linked to increased vulnerability to respiratory infections.

Structural challenges in the healthcare system such as uneven access to care, resource duplication, and inefficiencies in testing infrastructure have also impacted Mongolia’s COVID-19 response [66]. These systemic weaknesses underscore the broader challenge of sustaining an effective public health response beyond initial containment phases.

#### 3.2.2. Surveillance Data and Outbreaks

Mongolia was among the first countries to act swiftly upon the news of the SARS-CoV-2 outbreak. With its proximity to China, the Mongolian government activated the State Emergency Committee in January 2020, even before the first national case was confirmed. This proactive move, based on its Disaster Protection Law, introduced multiple public health measures including the suspension of educational institutions, banning of mass gatherings, and travel restrictions, all of which contributed to delaying the local introduction of COVID-19 cases until March 10, 2020 [58].

By maintaining strict surveillance at border checkpoints and implementing self-isolation and quarantine measures, Mongolia was able to avoid community transmission until November 2020. This early containment was credited to rapid national coordination and clear public communication strategies. Intensive surveillance efforts included the deployment of thermal screening and symptom checks at airports and land borders, which were vital in reducing the importation risk during the early stage [58].

1. National seroprevalence surveillance efforts

In response to the increasing global spread of COVID-19, Mongolia conducted one of its earliest national surveillance studies from October to December 2020. This cross-sectional survey sampled 5000 individuals using age-stratified selection and measured SARS-CoV-2 IgG antibody levels to determine the extent of prior infection in the population. The findings indicated an extremely low crude seroprevalence rate of 1.4%, suggesting that community transmission had not occurred widely prior to the country’s first outbreak in late 2020. Test-adjusted estimates supported this conclusion with seroprevalence between 1.4 and 1.5% [57].

These results aligned with the nation’s minimal reported cases and reinforced the effectiveness of early non-pharmaceutical interventions. The absence of significant associations between demographic or geographic variables and seropositivity also suggested uniform success in limiting the spread nationwide at that time [57].

To understand the evolving epidemiological landscape, Mongolia initiated a longitudinal seroepidemiological study between October 2020 and December 2021 that spanned four rounds of data collection [59]. This study was aligned with the World Health Organization’s Unity Studies protocol and analyzed over 4000 follow-up participants, providing continuous insights into the virus’s spread and the public health response.

The surveillance data revealed a sharp rise in seroprevalence, from 1.5% in late 2020 to over 82% by the end of 2021. Importantly, it was found that by late 2021, 62.4% of the population had received vaccinations while among the unvaccinated, more than 64% had already been infected [59]. These findings underscore the scale of the epidemic that unfolded in 2021, particularly as containment measures were gradually lifted and new variants were introduced.

The study also highlighted occupational and demographic risk factors for infection, with males and individuals aged 20 and older demonstrating significantly higher odds of seroconversion. Health workers consistently showed elevated risk across all rounds of observation, indicating the importance of occupational exposure and the need for targeted interventions in frontline populations [59].

2. Genomic surveillance

The need to track circulating viral lineages prompted a national effort in molecular surveillance. Given the limited genomic sequencing infrastructure in Mongolia, a retrospective molecular study using Nanopore sequencing was conducted between November 2020 and October 2021 [70]. Out of nearly 5000 nucleic acid tests, 799 samples underwent whole genome sequencing.

The findings revealed that the 20B (B.1.1.46) lineage predominated in the initial local transmissions. Alpha and Delta variants appeared later and circulated more slowly compared to global trends. Notably, Beta and Gamma variants were not detected during the study period, which might reflect the effectiveness of public health restrictions that limited variant introductions [70]. This study highlighted the country’s nascent but expanding genomic capacity and emphasized the need for sustained investments in sequencing infrastructure to monitor emerging viral threats effectively.

Following an initial successful vaccination campaign that began in February 2021, a marked surge in COVID-19 cases was observed by June 2021, peaking at approximately 3000 cases daily [60].

The Alpha variant accounted for 94.6% of these infections, while the Delta variant was present in 5.4%. Despite high vaccine uptake, many vaccinated individuals were still susceptible to infection, particularly those who received inactivated or adenovirus-vectored vaccines. The results pointed to the mismatch between vaccine-induced immunity and the circulating variants, further aggravated by the relaxation of social distancing policies in mid-2021 [60]. This evidence clearly illustrates the limitations of immunity acquired through vaccines alone in controlling outbreaks, especially without the support of sustained surveillance and adaptive policies.

3. Surveillance capacity

Although not traditional virological surveillance, air quality monitoring during lockdowns offered indirect insight into population behavior and compliance. Satellite and terrestrial air monitoring data collected between November 2020 and February 2021 indicated substantial reductions in nitrogen dioxide, PM10, and PM2.5 levels, suggesting that population movement and industrial activity had significantly decreased during public health lockdowns [67]. The findings underscore the value of integrating environmental and public health surveillance systems to monitor the effectiveness of behavioral interventions.

4. National diagnostic preparedness

The limited laboratory capacity in Mongolia must be recognized as a critical issue for national preparedness. The absence of high-throughput viral genome sequencing, real-time pathogen detection, and multiplex diagnostics limits Mongolia’s ability to monitor virus evolution and emergence. This vulnerability was reflected in the delayed identification of Alpha and Delta variants during the 2021 surge [60,70]. Incorporating these findings, Mongolia must develop a strategic biodefense policy that includes the creation of virus characterization laboratories equipped to detect unknown respiratory pathogens. Investments in bioinformatics, pathogen discovery, and early warning systems are essential to reducing dependence on external testing and diagnostics.

#### 3.2.3. Public Health Interventions

1. Initial government actions and preparedness

Mongolia’s early response to the COVID-19 pandemic was swift, decisive, and grounded in prevention. Recognizing the threat posed by its geographic proximity to China, the Government of Mongolia activated the State Emergency Committee as early as January 2020, months before the first domestic case was detected. This action was taken in accordance with the 2017 Disaster Protection Law, which enabled the government to implement sweeping control measures. These included the closure of educational institutions, restrictions on public gatherings, and nationwide campaigns for personal hygiene such as handwashing and mask-wearing [58]. These early efforts delayed the first confirmed case until March 2020 and effectively prevented intensive care admissions or fatalities until July of the same year. Notably, Mongolia reported no community transmission up to November 2020, highlighting the success of early non-pharmaceutical interventions [57,58].

2. National surveillance and mass testing.

To track viral spread and enable targeted interventions, Mongolia initiated several national-level testing strategies. The “One door one test” campaign in February 2021 was the most visible initiative. Conducted during a lockdown in the capital city of Ulaanbaatar, this mass testing campaign sought to test one adult from each household using PCR assays. Although intended to provide rapid surveillance and facilitate reopening, the campaign identified only 131 cases out of hundreds of thousands tested and was later criticized for being methodologically flawed and resource-inefficient [66].

3. Vaccine procurement

Mongolia began its national COVID-19 vaccination campaign in February 2021, offering four types of vaccines: two adenovirus-vectored (Oxford AstraZeneca and Sputnik V), one inactivated virus vaccine (Sinopharm), and one mRNA vaccine (Pfizer BioNTech). By mid-August 2021, 62.7 percent of the population had been fully vaccinated. The government prioritized rapid vaccine distribution and established partnerships with international agencies and manufacturers to secure sufficient doses. Serological studies show that the immunogenicity of the vaccines varied significantly by type. Participants vaccinated with the mRNA vaccine demonstrated the highest levels of neutralizing antibodies, followed by those who received adenovirus-based and inactivated vaccines. Despite a successful initial rollout, the surge in cases in mid-2021 revealed gaps in protection, particularly among those vaccinated with inactivated vaccines. This prompted discussions around the need for booster doses and the strategic use of more potent vaccine platforms [60,62,68].

A notable aspect of Mongolia’s intervention strategy was its reliance on community-based organizations such as the Mongolian Red Cross Society. This group played a pivotal role in promoting public health messaging, distributing educational materials, and organizing volunteer activities during both the influenza and COVID-19 responses [71]. Their preparedness planning, initially focused on seasonal influenza, was effectively adapted to the COVID-19 response and helped disseminate vital information on non-pharmaceutical interventions such as self-isolation, mask use, and hand hygiene. By leveraging a network of over 6000 volunteers, the Red Cross contributed to public awareness and psychosocial support efforts. More than 2000 volunteers provided services such as grocery support, mental health check-ins, and public education to over 290,000 people and repatriated nationals in quarantine [71]. This grassroots approach demonstrated the effectiveness of community-led interventions, especially in a country with a dispersed rural population and limited medical infrastructure.

Healthcare professionals bore a significant burden during Mongolia’s pandemic response. Long working hours, high exposure risk, and resource limitations led to a notable prevalence of depression, anxiety, and stress among frontline workers. A nationwide study of 965 healthcare professionals reported that over 70% experienced anxiety symptoms, over 50% reported depressive symptoms, and more than 35% experienced stress [64].

These findings have direct implications for occupational health policy. Strengthening mental health services, reducing burnout, and promoting workplace wellness should be central components of any comprehensive intervention strategy. The study also emphasized the importance of self-efficacy and sleep quality in buffering psychological distress, offering actionable insights for human resource planning in future health emergencies [64].

Another study of Mongolian healthcare workers found that 93.7% supported mandatory occupational vaccination, reflecting both high trust in public health measures and a collective sense of responsibility. However, some hesitation was observed among younger staff and those working at tertiary hospitals, underlining the importance of tailored communication strategies within medical institutions [65].

4. Vaccine hesitancy

Vaccine hesitancy posed a challenge to Mongolia’s otherwise rapid vaccination effort. A cross-sectional survey of 2875 participants found that social media was a major source of vaccine misinformation, and reliance on it correlated with higher levels of hesitancy [69]. Older individuals, those with higher education, and those who received information from official government sources were more likely to accept vaccination. These results support the importance of using credible channels for vaccine communication. The study also identified that concerns over side effects and vaccine type were among the main reasons for reluctance [69]. To address these issues, Mongolia must strengthen its official outreach, particularly among rural and lower-educated populations, by delivering accessible information on vaccine safety and efficacy.

5. Environmental and economic observations

The lockdown periods had additional public health impacts, including changes in environmental quality. Satellite and ground monitoring during November 2020 to February 2021 recorded significant decreases in air pollutants such as nitrogen dioxide, PM10, and PM2.5, which are known contributors to respiratory disease [67]. This reduction in pollution levels suggests that lockdowns offered short-term respiratory health benefits, albeit indirectly.

#### 3.2.4. Impact upon Mongolia’s Animal Population

1. Domestic animals

No documented cases in Mongolia.

2. Wild animals

SARS-CoV-2 infection was confirmed in Eurasian beavers (*Castor fiber*) kept in a conservation facility in Ulaanbaatar, Mongolia. Among the 48 samples collected from symptomatic and deceased beavers, 46 tested positive for SARS-CoV-2 RNA. Additionally, 15 out of 23 serum samples tested antibody-positive, confirming exposure and seroconversion in the population [72]. This outbreak resulted in the death of two animals and respiratory illness in others, demonstrating the virus’s capacity to affect captive wildlife populations in Mongolia [72].

#### 3.2.5. Epidemiology

The beaver outbreak began shortly after a farm employee was diagnosed with COVID-19. Subsequent viral transmission from human to beavers and then among animals was confirmed through serologic and molecular testing. One asymptomatic beaver tested antibody positive, indicating subclinical transmission potential. The confined housing of animals in shared indoor space likely contributed to rapid viral spread. By contrast, the study of 200 Bactrian camels (*Camelus bactrianus*) in Umnugovi and Dundgovi provinces found evidence of bovine-like coronaviruses but essentially no evidence of MERS-CoV antibodies [17].

#### 3.2.6. Virus Types and Subtypes Relevant to Mongolia

Sequencing of five SARS-CoV-2 positive beaver samples identified the B.1.617.2 lineage, known as the Delta variant. These sequences showed close genetic similarity to human isolates circulating in Mongolia during April to September 2021. However, unique amino acid mutations were identified in ORF1a and ORF1b, including S2500F, A3657V, H604Y, and T1404M. These specific combinations were not previously recorded in human samples from Mongolia, suggesting the possibility of intra-animal viral evolution [72]. The camel study detected no MERS-CoV, and Bactrian camels showed high reactivity to bovine coronavirus, which is endemic but not not thought to be zoonotic [17].

1. Surveillance data and outbreaks

The SARS-CoV-2 investigation in beavers was carried out using RT-PCR, ELISA serology, and full genome sequencing in a biosafety level 3 facility in Ulaanbaatar. This remains the only known coronavirus surveillance event in wildlife within Mongolia to date. Although these studies represent important efforts, both were geographically limited and cross-sectional, providing only a snapshot rather than continuous surveillance.

2. Animal health interventions

In the beaver outbreak, no structured interventions such as quarantine, culling, or environmental decontamination were reported. The study’s findings emphasized the need for improved active surveillance and biosecurity in captive animal populations. Similarly, the camel study was purely investigative and did not result in any policy change or intervention. These findings highlight Mongolia’s limited operational capacity to detect and manage coronavirus infections in animal populations [17,72].

### 3.3. Pneumoviridae

Viruses from the Pneumoviridae family are enveloped, including negative-strand RNA viruses, and were defined as a separate independent family from Paramyxoviridae in 2016 [73]. This family consists of two genera: Orthopneumovirus and Metapneumovirus. Metapneumovirus includes two species, Avian metapneumovirus and Human metapneumovirus, infecting mammals or birds. Despite that, Orthopneumovirus consists of three species: Bovine orthopneumovirus, Human orthopneumovirus, and Murine orthopneumovirus, infecting mammals [73,74]. Transmission mainly occurs by aerosol droplets and physical contact [73]. Among these viruses, RSV is the most widespread in Mongolia; it depends to the species of Human orthopneumovirus. RSV is classified as two antigenic subtypes, RSV A and RSV B [75,76,77]. RSV is a substantial cause of acute respiratory infections, including lower respiratory tract infections (LRTIs) [78]. RSV was first identified in 1957 as the primary cause of bronchiolitis [79]. Some studies have shown that RSV negatively affects the respiratory, cardiovascular, digestive, and central nervous systems [80]. Also, RSV usually occurs in winter or spring [81,82].

#### 3.3.1. Impact of RSV on Mongolia’s Human Populations

In a remote district of Ulaanbaatar city, RSV created severe illness, leading to hospitalization in infants who are less than six months old. Also, RSV can affect all infants, regardless of whether the infants are born healthy and full-term [34]. RSV plays a role in spreading acute lower respiratory infections in Mongolia and increases the health burden. RSV incidence was strongly positively correlated with the volume of measles cases, and researchers have also highlighted that coinfection with RSV and measles may increase the risk of death in young children [18]. RSV has a significant negative impact on the health of mothers and children, especially infants, so special attention needs to be paid to this age group.

#### 3.3.2. Epidemiology

Young age significantly influences RSV infection (OR = 0.9). The following factors may affect RSV but are not statistically significant: attending daycare (OR = 1.41), current breastfeeding (OR = 1.3), pneumococcal carriage (OR = 0.9), number of total people in the household (OR = 0.9), and fuel type (OR = 0.8) [18]. In another study, eight-week-old infants were at higher risk of RSV infection and severe acute respiratory infection (SARI) [34]. Compared to influenza, RSV tends to occur before the weather gets too cold. Earlier than the COVID-19 pandemic, RSV usually occurs between October and April, reaches a high point in December/January, and finishes in April. However, after the pandemic, RSV cases significantly decreased [18]. Another study found that RSV activity tends to be highest in the winter months of each year, and that RSV season begins 6–9 weeks before influenza season [83]. The researchers noted a marked relationship between RSV season and cases of severe/very severe lower respiratory tract infections and between RSV and periods of radiologically confirmed pneumonia. Although the concentration of Streptococcus pneumoniae showed a positive association with RSV burden among the children with and without S. pneumoniae, statistically significant differences were not identified in the prevalence of RSV cases [18].

#### 3.3.3. Virus Types and Subtypes

According to a study by Lien Anh Ha Do et al. [18], conducted in Mongolia, which enrolled 5705 children under 2 years of age, RSV was 37.0% (*n* = 2113). Of these positive cases, 55.3% (*n* = 1169) were RSV A, 42.9% (*n* = 907) were RSV B, and 1.8% (*n* = 37) were co-infected with both RSV A and B. During the study, RSV B was the most common subtype from October 2016 to February 2017 and from October 2017 to February 2018. Moreover, in prospective and observational open cohort studies, along with the ILI and severe acute respiratory illness infants (*n* = 269), 9.3% (*n* = 25) were positive for RSV [34]. We are faced with the need to conduct in-depth research into the types of viruses circulating in Mongolia.

#### 3.3.4. Surveillance Data and Outbreaks

**Figure 4:**
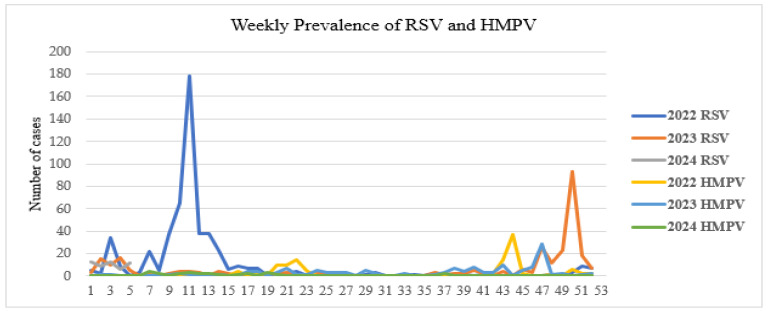
This graph presents the weekly prevalence of RSV and HMPV cases between 2022 and 2024. In 2022, a total of 6447 specimens were collected and tested from outpatient settings, and in 2023, a total of 8134 outpatient specimens were analyzed. The number of cases shown in the figure represents the findings detected through more than 100 outpatient-based units and clinics participating in the influenza surveillance network. However, it is not possible to determine whether the specimens were specifically collected from individuals with ILI, nor how many specimens were tested for RSV in detail. The testing method is also unspecified. In Mongolia, RSV activity peaks during the 11th week (spring) and again around the 50th week (winter). These data were sourced from the National Influenza Surveillance Office and have been available only since 2022.

No studies have been conducted on outbreaks of these viruses in Mongolia, and seasonal outbreaks are usually recorded annually. The RSV outbreak reached its initial peak in epidemiological week 11 of 2022 (Figure 4) and has exhibited a declining trend thereafter. A subsequent peak was observed in week 50 of 2023. In contrast, nationwide outbreaks of HMPV have been relatively infrequent, with a noticeable increase in cases observed in epidemiological week 44 of 2022 (Figure 4). Nevertheless, the number of HMPV cases remained considerably lower compared to RSV.

#### 3.3.5. Public Health Interventions

Researchers believe that measles-controlling strategies may be a way to prevent RSV infection [18]. A study was conducted in Mongolia to test the effectiveness of pneumococcal conjugate vaccine-13 against RSV infection. The study showed that the vaccine’s effectiveness varied between districts in Ulaanbaatar, Mongolia’s capital, but the results were not statistically significant [84]. There is a lack of research investigating the impact of RSV on the population. Its association with measles infection could be considered in future policy and prevention planning, and public health interventions can be planned. There is also a pressing need to increase laboratory capacity to detect viruses more accurately, upgrade diagnostic reagents, and improve preparedness during outbreaks.

#### 3.3.6. Animal Diseases

No animal studies related to this virus family appear to have been conducted in Mongolia. An exhaustive search of multiple online sources yielded no relevant results, including PubMed, Google Scholar, ProQuest, ScienceDirect, Springer Nature, Virology Journal, The Journal of Infectious Diseases, and The Lancet.

In several countries, *bovine respiratory syncytial virus* has been reported in some countries to infect and cause disease not only in cattle but also in horses [85,86], suggesting that a similar phenomenon may be occurring in Mongolia. In addition, *avian metapneumoviruses* are known to infect both wild birds and chickens [87,88,89], and such infections are likely to occur among bird populations in Mongolia.

### 3.4. Adenoviridae

The family *Adenoviridae* includes six genera and a hundred-twenty-five species [90]. Among these genera and species of the *Adenoviridae* family, only *Human adenoviruses* have been studied in Mongolia. *Human adenoviruses* (HAdV) belong to the *Mastadenovirus* genera. They infect mammals, are segmented into sixty-seven serotypes and seven subgroups (A to G), have double-stranded DNA, and are non-enveloped. These serotypes and subgroups cause respiratory infections and gastrointestinal inflammation in humans [91]. HAdV can infect people of any age but is most common in the pediatric age group, especially young children and infants. By age 10, most children have had at least one HAdV infection [92]. Although outbreaks typically occur in the winter and early spring [93,94,95,96], transmission occurs year-round and has no clear seasonal pattern [97,98]. HAdV is transmitted from human to human through airway secretions, the fecal–oral route, and through contaminated surfaces [99]. Hospital-acquired infections can also occur in healthcare facilities due to workers and poorly sterilized equipment [99]. Transmission can also happen through external sources (e.g., pillows, bedding, cabinets, etc.) [100,101] or reactivation [102].

#### 3.4.1. Impact of HAdV on Mongolia’s Human Population and Epidemiology

Since HAdV-D8 is associated with epidemic keratoconjunctivitis (EKS) [103,104,105], HAdV also contributes to the spread and cause of EKS in Mongolia. The four seasons of the year are not a risk factor for HAdV in Mongolia. Nevertheless, researchers noted that many positive cases of HAdV were reported from September to December 2010. Additionally, HAdV causes 2.1% of ILI [106].

#### 3.4.2. Virus Types and Subtypes

In Mongolia, from January 2010–May 2011, randomly selected 1950 influenza-negative patients with ILI samples were tested for HAdV. As a result of multiplex reverse transcription polymerase chain reaction (RT-PCR) or immunofluorescence assay, 2.1% (*n* = 40) were positive for HAdV. On the other hand, in a phylogenetic analysis, 31 samples were positive for the hexon genes. Besides that, in 31 HAdV-positive samples with hexon genes, seven serotypes were identified; the most common serotypes were HAdV-B7, HAdV-B3, and HAdV-D8. Also, the following serotypes have been detected in Mongolia: HAdV-C1, -C2, -C5, -C6. However, there were no meaningful statistical differences in the frequency of these serotypes. The nucleotide sequences of HAdV-B7 and HAdV-B3 serotypes also matched, implying that they may have been transported to Mongolia from other countries more than 10 years ago [106]. The fact that the B7 strain of HAdV, which has high virulence, prevalence, and mortality rates, is the most prevalent in Mongolia is a significant issue, and constant monitoring, research, and detection are essential.

#### 3.4.3. Surveillance Data and Outbreaks

**Figure 5:**
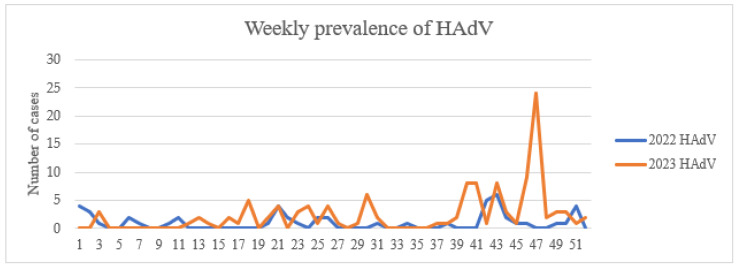
This graph presents the weekly prevalence of Human Adenovirus (HAdV) in Mongolia during 2022 and 2023. The number of cases shown in the figure represents the findings detected through more than 100 outpatient-based units and clinics participating in the influenza surveillance network. However, it is not possible to determine whether the specimens were specifically collected from individuals with ILI, nor how many specimens were tested for HAdV in detail. The testing method is also unspecified. These data were obtained from the National Influenza Surveillance Service for the years 2022–2023.

No studies have been conducted on epidemics of this virus in Mongolia at present, and seasonal outbreaks are usually recorded annually. HAdV outbreaks peaked in epidemiological week 47 of 2023 and have shown a declining trend since then (Figure 5). Although relatively few outbreaks were recorded in 2022, a higher number of cases was observed in week 43 compared to other weeks.

#### 3.4.4. Public Health Interventions

Researchers highlight the need to regularly monitor HAdV serotypes detected in Mongolia and conduct further extensive studies. In addition to publishing the virus’s distribution and characteristics in domestic journals, researchers’ capacity must be significantly increased, and evidence must be compiled and published in international journals.

#### 3.4.5. Animal Diseases

No animal studies related to this virus family appear to have been conducted in Mongolia. An exhaustive search of multiple online sources yielded no relevant results, including PubMed, Google Scholar, ProQuest, ScienceDirect, Springer Nature, Virology Journal, The Journal of Infectious Diseases, and The Lancet.

In some countries of the world, *equine adenovirus* 1 has been reported in horses [107,108,109], and *fowl adenovirus* are known to infect avian species such as chickens [110,111], therefore the potential for infection in Mongolian horse and bird populations warrants consideration and further investigation. Moreover, *canine adenovirus* has also been identified as a pathogen infecting dogs [112,113], underscoring the importance of considering this virus in diagnostic evaluations and emphasizing the need for further research.

### 3.5. Paramyxoviridae

The *Paramyxoviridae* family includes large, enveloped RNA viruses that infect mammals, birds, and, in some cases, reptiles and fish. The *Paramyxoviridae* family currently includes four subfamilies, 20 genera and 78 species. These viruses are transmitted primarily through direct contact and airborne routes, with no known vectors [114]. HPIV causes infections of the lungs and respiratory tract. It can also cause serious illnesses in children, such as croup, bronchitis, bronchiolitis, and pneumonia [115]. Several investigations conducted in Mongolia have reported the detection of Newcastle disease virus (NDV; APMV-1), as well as Avian Paramyxovirus types 4 and 8 (APMV-4 and 8) and Peste des petits ruminants virus (PPRV) in domestic poultry, wild migratory waterfowl and wild ungulates [116,117]. The presence of pathogens with zoonotic properties raises concerns that known and not previously recognized paramyxoviruses could cause epidemics [118].

#### 3.5.1. Impact upon Mongolia’s Human Populations

Respiratory viruses other than influenza are more frequently observed in children under five years of age [115]. HPIV is a significant cause of SARI as well as ILI, and is therefore a leading contributor to hospitalization and morbidity in this age group [119].

1. Epidemiology in human populations

Surveillance studies conducted between the 2007/08 and 2013/14 seasons demonstrated that human parainfluenza virus (HPIV) infections occurred predominantly outside the peak influenza season, with notable activity observed at 10–12 months and 3–5 months of the year. HPIV may contribute to the seasonal increase in both ILI and SARI. Between 2010 and 2013, HPIV was detected in both ILI and SARI cases, although at lower rates compared to HRV, RSV and HMPV. While HPIV did not account for the majority of cases, a peak in SARI incidence was observed during the 2010/11 season, coinciding with a seasonal rise in ILI. Unlike HRV, which circulates year-round, HPIV-3 has demonstrated an intermittent circulation pattern [119].

The study also found that, unlike RSV and MPV, which predominantly affect infants and young children, HPIV infection exhibited an age-specific distribution. Most SARI cases associated with HPIV occurred in children under one year of age; however, a higher frequency of SARI was also observed among individuals aged 45–64 years. These findings suggest that older adults and potentially immunocompromised individuals may be at increased risk of severe HPIV-associated illness [119].

2. Virus types and subtypes relevant to Mongolia

HPIV is classified into four subtypes (HPIV-1 to HPIV-4), with HPIV-3 reported as the most prevalent subtype globally [120]. In Mongolia, only HPIV-3 has been identified to date, and no data are currently available regarding the circulation of the other three subtypes [27,119].

3. Surveillance data and outbreaks

Although Mongolia’s respiratory virus surveillance system primarily targets influenza, available data indicate that individuals presenting with ILI and SARI have also been tested for other respiratory viruses, including HRV, MPV, HPIV, and RSV [27,119].

In this surveillance study, a total of 1350 respiratory samples that tested negative for influenza virus during the 2010/11 to 2013/14 seasons were selected for further testing for other respiratory viruses. Of these, 563 samples (41.7%) were positive for at least one virus. RV was the most frequently detected (24.7%), followed by HMPV (11.4%), RSV (8.0%), and HPIV-3 (5.5%), which showed the lowest detection rate. The highest prevalence of HPIV-3 was observed in children under one year of age (3.4%). Among all ILI cases, HPIV-3 was detected in 2.3% [119].

A total of 1682 SARI cases that tested negative for influenza virus during the 2010/11 to 2013/14 seasons were further analyzed for other respiratory viruses. Among these cases, 763 (45.4%) were positive for at least one virus. HRV was detected in 19.0%, RSV in 18.7%, HMPV in 11.1%, and HPIV-3 in 5.9% of cases. HPIV-3 accounted for 2.7% of all SARI cases. The prevalence of HRV, RSV, and HMPV was highest in children under four years of age, whereas HPIV-3 showed the highest prevalence among adults aged 45–64 years, reaching 4.7%. The peak detection rate for HPIV-3 in SARI cases occurred in 2010, at 5.2%. While HRV and RSV remain the predominant causes of SARI, HPIV-3 contributes to a notable proportion of cases [119].

An epidemiological study in Mongolia demonstrated that the incidence of ILI remained elevated following the decline of influenza activity in the 2010–2011 season. Analysis of a random subset of 606 ILI samples revealed significant detection rates of HRV, RSV, and HMPV, with HPIV-3 identified in 7% (19 samples) of cases [27]. These findings suggest that HPIV-3 is a contributing pathogen to ILI in Mongolia.

The increased incidence of ILI observed before and after the peak influenza virus circulation may be attributed to other respiratory viruses, including HRV, RSV, HMPV, and HPIV-3.

4. Public health interventions

In Mongolia, the influenza response and surveillance system has been progressing steadily [27]. However, the detection of HPIV in samples originally collected for influenza surveillance but testing negative highlights gaps in respiratory virus detection and diagnosis outside of influenza peak periods [119]. The limited data on HPIV circulation further suggests that current surveillance efforts do not comprehensively cover this virus, underscoring the need for expanded and more inclusive respiratory pathogen monitoring.

Although HPIV is not among the leading causes of respiratory illness, regular surveillance is warranted due to its ability to cause both mild and severe disease. Ongoing monitoring will facilitate timely diagnosis and management, particularly for vulnerable populations such as infants and the elderly.

#### 3.5.2. Impact upon Mongolia’s Animal Population

APMV-1 is a zoonotic avian pathogen that causes severe outbreaks with high morbidity and mortality, especially in velogenic strains, which can cause 70–100% mortality in unvaccinated flocks. Clinical manifestations include respiratory distress, neurological signs, decreased egg production, and sudden death [121].

APMV-4 and APMV-8 cause mild respiratory disease in wild and migratory birds. APMV-4 and APMV-8 are considered zoonotic viruses, meaning they can be transmitted from animals to humans [122]. However, although several cases have been found in wild migratory birds in Mongolia [116], there are no reports of human transmission.

Virological research on wild bird populations in Mongolia has predominantly concentrated on avian influenza viruses, with limited attention given to other avian pathogens, resulting in a significant gap in comprehensive avian disease surveillance

PPRV is a highly contagious and often fatal viral disease primarily affecting small ruminants. Clinical manifestations include fever, oral erosions, and respiratory complications such as pneumonia [123].

The virus spreads rapidly among grazing sheep and goats, leading to outbreaks that cause substantial economic losses for pastoralist communities due to livestock mortality, decreased productivity, and trade restrictions. During the 2016–2017 outbreak, mortality rates reached up to 80% among the infected population [117,124].

PPRV has also been reported to infect wild ungulate species, particularly saigas (*Saiga tatarica*), argali (*Ovis ammon*), gazelles (*Gazella* spp.), and ibex (*Capra* spp.), often resulting in high mortality. In 2017, a severe outbreak led to the death of over two-thirds of the critically endangered Mongolian saiga population [125]. This mass mortality event not only presents a significant threat to species conservation but also disrupts the ecological balance of the steppe ecosystem [126].

1. Epidemiology in animal populations

**Figure 6:**
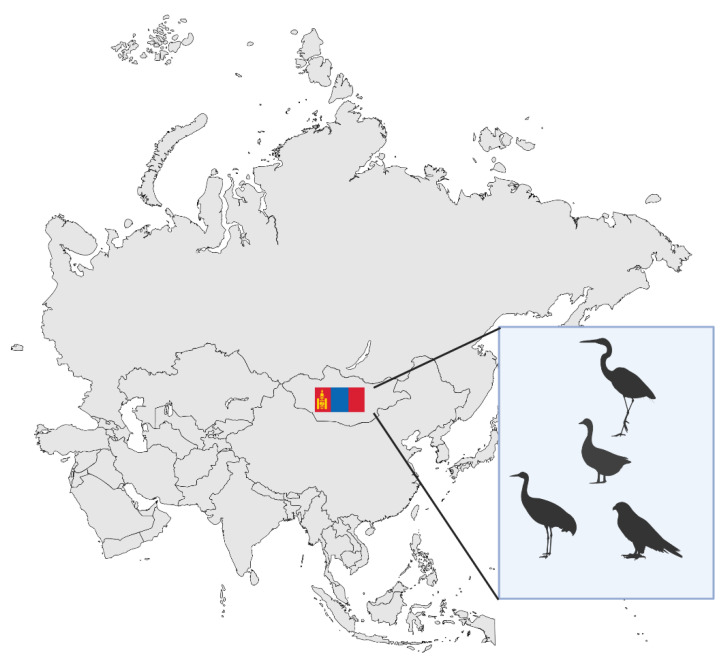
Mongolia as a migratory pathway for several bird species. Mongolia’s distinctive geographic and ecological features make it an ideal setting to investigate the epidemiology of avian paramyxoviruses (APMVs) in wild bird populations. The country lies at the intersection of four major migratory flyways—East Asia/Australasia, Central Asia/India, West Asia/Africa, and the Mediterranean/Black Sea—placing it at a strategic crossroads for studying avian movement and pathogen transmission. An estimated 391 migratory bird species pass through or breed in Mongolia, which functions as a vital stopover, refueling site, and breeding ground for diverse species, including cranes, geese, egrets, eagles, and falcons. Created in BioRender. Trujillo Vargas, C. (2025) https://BioRender.com/6r2ncj1.

Mongolia’s unique combination of geographic and ecological characteristics provides an optimal setting for investigating the epidemiology of avian paramyxoviruses (APMVs) in wild bird populations. Mongolia is situated along four main migratory routes, East Asia/Australasia, Central Asia/India, West Asia/Africa, and the Mediterranean/Black Sea, positioning it as a key region for studying avian movement and the epidemiology of associated pathogens. Approximately 391 species of migratory birds are known to pass through or breed in Mongolia (Figure 6) [121].

APMVs are primarily transmitted via direct contact with infected birds, exposure to respiratory secretions, or through fomites, including contaminated feed, water, and equipment [122]. The geographic location along this migratory bird route increases the risk of infection.

PPRV is endemic in most African countries; however, in recent years, its presence has also been confirmed in Central Asia, Southeast Asia, and the Middle East [125].

Mongolia reported its first outbreak of PPRV in September 2016, when mortality in domestic sheep and goats was traced to the introduction of the virus from neighboring China. Later that year, PPRV was confirmed for the first time in the saiga antelope (*Saiga tatarica mongolica*), a critically endangered subspecies [117,124].

The distribution range of the Mongolian saiga partially overlaps with that of mountain-dwelling ungulates such as the Siberian ibex (*Capra sibirica*) and argali sheep (*Ovis ammon*), as well as plains-dwelling species such as the goitered gazelle (*Gazella subgutturosa*) and the Mongolian gazelle (*Procapra gutturosa*). This range also coincides with the core habitat of the saiga [18], where over 1.5 million domestic sheep and goats graze seasonally across both mountainous and steppe ecosystems [117]. Such overlap facilitates the transmission of PPRV, which is endemic in small livestock, to susceptible wild ungulate populations.

2. Virus types and subtypes relevant to Mongolia’s animal population

In 2012, a surveillance study in Mongolia detected avian paramyxovirus (APMV) during routine monitoring for avian influenza viruses. In a study by Erdene-Ochir et al., APMV-4 and APMV8 serotypes were identified among 1907 samples collected from wild migratory waterfowl. Phylogenetic analysis of the isolates revealed close clustering with Asian APMV-4 and APMV-8 strains recently detected in wild birds from Korea, Japan, China, and Kazakhstan. DNA barcoding indicated that the Mongolian strains were isolated from Anseriformes, including Mallards and Whooper Swans [116].

PPRV can be classified into four genetically distinct lineages through phylogenetic analysis of the nucleocapsid (N) gene. Lineages I, II, and III are predominantly confined to Africa, whereas Lineage IV is primarily distributed across the Middle East, China, and other parts of Asia [127].

In 2016, pathological samples collected from sheep and goats infected with PPRV in western Mongolia were subjected to gene sequencing and phylogenetic analysis, which confirmed the presence of PPRV belonging to Lineage IV.

In 2017, phylogenetic analysis of samples from two PPRV-infected saiga antelopes confirmed that the virus belonged to Lineage IV. These findings provide molecular evidence supporting the transmission of PPRV from domestic livestock to wild ungulates [117].

Analysis of the complete RNA genome of the virus revealed a high degree of similarity (99.0–99.5%) to PPRV strains circulating in China between 2013 and 2015. The virus was identified as originating from an outbreak in Xinjiang, a northwestern region of China that shares a border with Mongolia. These findings support the hypothesis of a common origin for the viruses and suggest that cross-border movement of animals may have facilitated the transmission of PPRV between the two countries [128].

This represents the first complete PPRV genome sequenced from Mongolia and is of significant importance for demonstrating the common origin of PPRV strains circulating in East Asia. The genomic data provide valuable insights for regional disease surveillance, control strategies, and transboundary management efforts [128].

3. Surveillance data and outbreaks

While no outbreaks of APMV have been documented in migratory bird populations [128], APMV-4 and APMV-8 were detected in wild waterfowl during surveillance studies conducted in 2012. In the autumn of 2016, a PPR outbreak occurred in western Mongolia, affecting 14 soums across three provinces. A total of 83,889 small ruminants from 1081 households were infected, resulting in 12,976 animal deaths, corresponding to a case fatality rate of 15.5%. Following the outbreak, approximately 10 million small ruminants in surrounding areas were vaccinated, effectively containing the spread of this highly contagious disease [117,129].

However, in late 2016, a sharp decline in the Mongolian saiga population was observed, which was subsequently confirmed to be caused by PPRV infection. Shortly thereafter, mortality due to the virus was also documented in Siberian ibex (*Capra sibirica*) and gazelle species. In the ensuing months, thousands of Mongolian saiga, already classified as critically endangered and listed in the national Red Book, succumbed to the disease [117,129].

According to population estimates, the Mongolian saiga population declined by approximately 80%, raising serious concerns about the species’ survival. The number of individuals dropped from 25,699 in January 2017 to 8806 in May 2017—the final month for which robust abundance modeling was available and coinciding with the end of the mass mortality event. However, the decline continued beyond this period. A follow-up survey conducted in April 2018, nearly one year after the outbreak, recorded a population of just 5142 individuals, representing only 20% of the January 2017 population [117].

In 2021, an outbreak of PPRV was detected in a mixed flock of sheep and goats in southern Mongolia near the border with China. Among 807 sheep grazing in the area, 40 individuals exhibited clinical signs consistent with PPR; however, no mortalities were reported. Diagnostic samples collected and analyzed at the State Veterinary Central Laboratory confirmed the presence of the PPRV genome. Following confirmation, the affected herd was promptly vaccinated and placed under quarantine. No additional PPR cases were reported after implementation of these control measures [126].

In December 2024, an outbreak of PPR was reported in Khovd Province. However, it was rapidly contained through the culling of infected animals, according to the World Organisation for Animal Health (WOAH).

4. Animal health interventions

Enhanced surveillance of avian populations, including domestic poultry and wild birds in Mongolia, would significantly advance the scientific understanding of the global distribution of Avian Metapneumovirus subtype 4 (AMPV-4) and Avian Paramyxovirus serotype 8 (APMV-8), as well as elucidate their transmission dynamics, evolutionary patterns, host range ecology, and epidemiological characteristics [116].

PPRV is a highly contagious viral disease of small ruminants, associated with high morbidity and mortality rates in over 70 countries. The disease is estimated to cause global economic losses of approximately USD 2.1 billion and adversely affects the livelihoods of around 900 million poor and low-income individuals. The implementation of effective, rapid, and coordinated control measures is therefore essential to prevent further spread and mitigate its socio-economic impact [130].

A global strategy has been established to eradicate PPRV by 2030, focusing on mass vaccination campaigns, enhanced surveillance, and international collaboration. In parallel, targeted programs are being implemented to protect susceptible wild ungulate populations, recognizing their ecological importance and vulnerability to PPRV transmission [130].

Mongolia is currently developing and implementing a ‘National Strategy for the Control and Eradication of PPR’ for the period 2022–2030. Within this framework, the country is actively contributing to global efforts aimed at the eradication of PPRV, while simultaneously working to strengthen national veterinary services and establish a comprehensive disease surveillance system. As part of this strategy, Mongolia is conducting targeted surveillance studies to detect PPRV circulation and carrying out vaccination campaigns to protect its livestock populations [131].

### 3.6. Picornaviridae

The *Picornaviridae* family consists of small viruses characterized by an icosahedral shape and features positive-sense single-stranded RNA, which can be immediately utilized for protein synthesis. This family of viruses comprises 63 genera and 147 identified species, with numerous others still under investigation [132]. The *Picornaviridae* family consists of numerous significant viruses that can impact both humans and animals, resulting in major health and economic issues [133]. Six genera of the *Picornaviridae* family include viruses that infect humans. These genera are responsible for a variety of diseases, ranging from the common cold to more serious conditions like polio and meningitis. The six genera of the *Picornaviridae* family that include pathogens infecting humans are *Enterovirus, Parechovirus, Hepatovirus, Cardiovirus, Kobuvirus*, and the proposed genus *Cosavirus* [134]. Despite limited available data on these viruses in Mongolia, few studies have documented the detection of coxsackievirus types A16, enterovirus types C117, D68, and EV71 [135,136,137].

Zoonotic transmission of viruses within the *Picornaviridae* family is Food-and-Mouth Disease virus. FMD virus was first identified in Mongolia in 2010 and has since been associated with several outbreaks among small livestock and wild ungulate populations [138,139].

#### 3.6.1. Impact upon Mongolia’s Human Populations

Rhinoviruses are the leading cause of the common cold and are a frequent viral trigger for asthma episodes [140].

#### 3.6.2. Epidemiology

Most rhinovirus infections result in either no symptoms or only mild symptoms. They can lead to serious illness, particularly in individuals with a weakened immune system, asthma, or other existing health conditions. Rhinoviruses spread throughout the year, but their activity typically increases in early fall and spring [140]. HRVs typically cause epidemics in early fall and late spring. In this study, HRV infections were detected year-round, with peaks in late summer and early fall. HRV-A was most common in late summer and early fall, HRV-B was most common in fall and winter, and HRV-C was most common in late spring, fall, and winter [141].

#### 3.6.3. Virus Types and Subtypes Relevant to Mongolia

The genomic structure of *Human Rhinoviruses* (HRVs) has been studied and shows that there are three different HRV groups, called groups A, B, and C, which belong to the *Enterovirus* genus in the *Picornaviridae* family. According to a study by Sosorbaram Tsatsral et al., HRV-A was detected in 47.1% of cases, HRV-C in 44.7%, and HRV-B in 8.2%. Between 2008 and 2013, HRV-A and HRV-C were the leading causes of hospitalization for respiratory diseases in Mongolia, with most HRV-positive patients infected with either HRV-A or HRV-C. HRV-B accounted for just 8.2% of cases [141].

#### 3.6.4. Surveillance Data and Outbreaks

In one study, 2689 nasopharyngeal swabs collected in Mongolia between 2008 and 2013 were analyzed, detecting human rhinovirus (HRV) in 295 samples (11.0%), of which 170 (57.6%) were subjected to further analysis [141].

#### 3.6.5. Public Health Interventions

Despite its high prevalence, HRV remains underprioritized in Mongolia, with no specific vaccines, treatments, or public health campaigns addressing it. These findings underscore the necessity of formulating evidence-based control strategies for influenza, informed by robust epidemiological and virological data. HRV is a major cause of acute respiratory infections in children under five, who are especially vulnerable due to Mongolia’s harsh climate and environmental risk factors.

## 4. Conclusions

We trust readers will fine this narrative review helpful in considering the many respiratory viruses that impact human and veterinary health in Mongolia. It is clear from this review that among the viral families discussed here most available data involve studies of viruses in the *Orthomyxoviridae* and *Coronaviridae* families. Data are relatively sparse regarding viruses in other families and subsequently there are large gaps in knowledge regarding our understanding of the human and animal morbidity associated with viruses in these viral families. Similarly, our understanding of transmission ecology for these viruses in these viral families is limited and often there are few therapeutics available. These gaps in data are important for Mongolian public health and veterinary health officials to consider.

**Figure 7:**
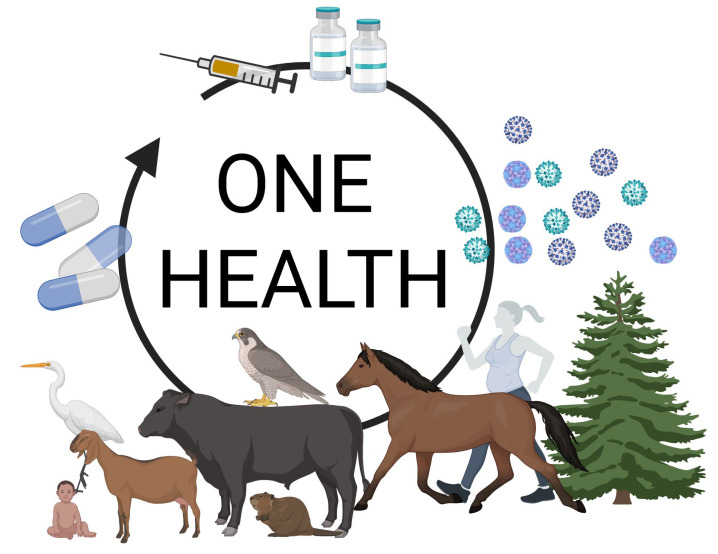
Beyond Influenza and SARS-CoV-2, many other viral pathogens remain underprioritized in Mongolia. Advancing integrated One Health research—linking active epidemiological surveillance across wildlife, livestock, and human populations with viral characterization and the development of vaccines and therapeutics—could help bridge these critical gaps. Such efforts would strengthen public health resilience and protect the most vulnerable groups, including young children, pregnant women, the elderly, and individuals with chronic conditions. This approach is particularly relevant across Mongolia’s diverse ecosystems—from steppe and desert regions to forested and high mountain zones—as well as among nomadic and semi-nomadic communities and within rapidly expanding urban centers, where human–animal–environment interactions are intensifying. Created in BioRender. Trujillo Vargas, C. (2025) https://BioRender.com/3fshm66.

While other disease groups such as cardiovascular diseases, cancer, alcohol use disorders, diabetes, diarrheal diseases, and nutritional deficits also cause significant morbidity in Mongolia, one can easily argue that respiratory virus morbidity is among the most important problems Mongolia faces today. The data in this review summarize specific viruses causing this morbidity and offer insight regarding how Mongolia chose to invest in future respiratory virus disease mitigation efforts. Such investments, especially employing a One Health approach (Figure 7), could greatly improve human and animal health metrics in Mongolia.

## Figures and Tables

**Table 1:** Virus types and subtypes identified in humans and animals in Mongolia.

Viral Family	Human Viruses	Animal Viruses
*Orthomyxoviridae*	Influenza A (H1N1), A (H1N1) pdm09, A (H3N2), Influenza B/Victoria, B/Yamagata	Equine influenza A (H3N8), reassortant H1N1 (camel), highly pathogenic avian influenza virus (H5N1, H5N6, H5N8), low pathogenic avian influenza subtypes (H1N1, H2N3, H3N6, H3N8, H4N6, H5N3, H6N1, H7N3, H7N7, H8N4, H10N7, H12N3, H13N6, H13N8, H16N3)
*Coronaviridae*	SARS-CoV-2	SARS-CoV-2, bovine-like CoV
*Pneumoviridae*	Respiratory syncytial virus (RSV A, RSV B, RSV A + B coinfection)	No animal studies or detections reported in Mongolia
*Adenoviridae*	Human adenoviruses (HAdV-B7, B3, D8, C1, C2, C5, C6)	No animal studies or detections reported in Mongolia
*Paramyxoviridae*	Human parainfluenza virus (HPIV-3)	Avian paramyxoviruses (APMV-1, APMV-4, APMV-8), Pesde des petits ruminants virus (PPRV)
	Human rhinoviruses (HRV-A, HRV-B, HRV-C)	Food-and-mouth disease virus

## Data Availability

No new data were created or analyzed in this study. Data sharing is not applicable to this article.

## Abbreviations

The following abbreviations are used in this manuscript:

WHO	World Health Organization
COVID-19	Coronavirus disease
SARS-CoV-2	Severe Acute Respiratory Syndrome Coronavirus 2
MERS-CoV	Middle East respiratory syndrome coronavirus
EIV	Equine influenza virus
BCoV	Bovine coronavirus
DNA	Deoxyribonucleic acid
HAdV	Human adenovirus
FAO	Food and Agriculture Organization of the United Nations
UNDP	United Nations Development Programme
UN-Habitat	United Nations Human Settlements Programme
HPIV	Human parainfluenza virus
ILI	Influenza-like-illness
SARI	Severe acute respiratory infection
RSV	Respiratory syncytial virus
RNA	Ribonucleic acid
HMPV	Human metapneumovirus
AIVs	Avian influenza viruses
LPAIVs	Low-pathogenicity avian influenza viruses
HPAIVs	Highly pathogenic avian influenza viruses
HPAI	Highly pathogenic avian influenza
ODOT	One door-one test
NDV	Newcastle Disease Virus
APMV	Avian Paramyxovirus
PPRV	Peste des Petits Ruminants Virus
